# Influence of Cobalt Source, Folic Acid, and Rumen-Protected Methionine on Performance, Metabolism, and Liver Tissue One-Carbon Metabolism Biomarkers in Peripartal Holstein Cows

**DOI:** 10.3390/ani13132107

**Published:** 2023-06-25

**Authors:** Vincenzo Lopreiato, Abdulrahman S. Alharthi, Yusheng Liang, Ahmed A. Elolimy, Ryan Bucktrout, Mike T. Socha, Erminio Trevisi, Juan J. Loor

**Affiliations:** 1 Department of Veterinary Sciences, University of Messina, Viale Giovanni Palatucci, snc, 98168 Messina, Italy; 2 Department of Animal Production, College of Food and Agriculture Sciences, King Saud University, Riyadh 11451, Saudi Arabia; 3 Department of Animal Sciences, Division of Nutritional Sciences, University of Illinois, Urbana, IL 61801, USA; 4 Animal Production Department, National Research Centre, Dokki, Giza 12622, Egypt; 5 Zinpro Corporation, Eden Prairie, MN 55344, USA; 6 Department of Animal Sciences, Food and Nutrition (DiANA), Faculty of Agriculture, Food and Environmental Science, Università Cattolica del Sacro Cuore, 29122 Piacenza, Italy

**Keywords:** methylation, lactation, methyl donors

## Abstract

**Simple Summary:**

Vitamin B_12_ is produced by ruminal microbes, and dietary Co is essential in this process, ultimately residing at the center of the corrin ring of the vitamin. Together with folate, vitamin B_12_ participates in the remethylation of homocysteine to generate methionine. Several studies with dairy cows have highlighted the positive effect of supplementing Co above current estimated requirements on the ruminal synthesis of vitamin B_12_. Studies with dairy cows have yielded inconsistent results in terms of lactation performance both in early- and mid-lactation phases. Despite this, it was clearly demonstrated that concentrations of Co and vitamin B_12_ in the liver rise markedly after calving when periparturient Holstein cows are fed supplemental Co. We sought to assess if Co source, folic acid, and rumen-protected methionine (RPM) fed in various combinations around parturition have an impact on performance and one-carbon metabolism in the liver. Despite a lack of effect on performance, feeding ruminally available folic acid with an experimental source of Co and RPM had the strongest effect on the activity of hepatic methionine adenosyltransferase, which produces the methyl donor S-adenosylmethionine. Thus, these nutrients may impact methyl-group-requiring reactions in the liver during the transition period.

**Abstract:**

Vitamin B_12_ plays a role in the remethylation of homocysteine to Met, which then serves as a substrate for Met adenosyltransferase (MAT) to synthesize S-adenosylmethionine (SAM). We investigated effects of feeding two cobalt sources [Co-glucoheptonate (CoPro) or CoPectin, Zinpro Corp.], an experimental ruminally-available source of folic acid (FOA), and rumen-protected Met (RPM) on performance and hepatic one-carbon metabolism in peripartal Holstein cows. From −30 to 30 d around calving, 72 multiparous cows were randomly allocated to: CoPro, CoPro + FOA, CoPectin + FOA, or CoPectin + FOA + RPM. The Co treatments delivered 1 mg Co/kg of DM (CoPro or CoPectin), each FOA group received 50 mg/d FOA, and RPM was fed at 0.09% of DM intake (DMI). Milk yield and DMI were not affected. Compared with other groups, the percentage of milk protein was greater after the second week of lactation in CoPectin + FOA + RPM. Compared with CoPro or CoPro + FOA, feeding CoPectin + FOA or CoPectin + FOA + RPM led to a greater activity of MAT at 7 to 15 d postcalving. For betaine–homocysteine S-methyltransferase, CoPro together with CoPectin + FOA + RPM cows had greater activity at 7 and 15 d than CoPro + FOA. Overall, supplying FOA with CoPectin or CoPectin plus RPM may enhance S-adenosylmethionine synthesis via MAT in the liver after parturition. As such, these nutrients may impact methylation reactions and liver function.

## 1. Introduction

Although trace elements are needed by the body in small amounts, they are essential nutrients for several metabolic functions, such as growth, reproduction, and immunity. Besides the easily understandable role of methionine (**Met**) [1,2,3,4], the one-carbon metabolism pathway relies strongly on the key enzyme 5-methyltetrahydrofolate-homocysteine methyltransferase (**MTR**), with vitamin B_12_ as a cofactor (cobalamin), and folate that participates in the transfer of one-carbon units to form 5-methyltetrahydrofolate, which then donates a methyl group to cobalamin (MTR complex as acceptor), forming methyl-cobalamin, and then to homocysteine, forming Met (remethylation) [5,6].

Vitamin B_12_ is produced by ruminal microbes, and cobalt (**Co**) is essential, residing at the center of the corrin ring of vitamin B_12_ [7]. Dietary Co content has been shown to positively influence ruminal vitamin B_12_ synthesis, increasing the efficiency from 3% with insufficient amounts of dietary Co up to 13% when, instead, adequate dietary Co is available [8]. A marked revision of dietary requirements for Co in dairy cattle was performed by the latest National Research Council edition (2021) [9], doubling the requirement from 0.11 to 0.20 mg/kg of dry matter (**DM**). This is the result of several studies that highlighted the positive effect of supplementing Co above 0.11 mg/kg of DM on the ruminal synthesis of vitamin B_12_ [10,11,12,13]. However, there are a certain number of studies with inconsistent results in terms of lactation performance both in early- and mid-lactation phases. Despite these inconsistent responses in terms of milk yield, it was clearly demonstrated that, in spite of the absence of effects for energy metabolism at the plasma level when periparturient Holstein cows are fed supplemental Co, the concentration of both Co and vitamin B_12_ in the liver increases markedly after calving [13]. 

As mentioned above, folic acid (**FOA**) is a B-complex vitamin that mediates the transfer of a methyl group in one-carbon metabolism. It was reported previously that the demethylation of 5-methyltetrahydrofolate is blocked if a lack of vitamin B_12_ occurs, reducing folate utilization at the cellular level [14]. Clearly, the metabolism of FOA and Met is the key for the production of S-adenosylmethionine (**SAM**), the main donor of methyl groups, and increasing the dietary supply of FOA could lead to greater endogenous Met synthesis [15]. The latter is supported by the study of Graulet et al. [16] with periparturient dairy cows, where supplements of FOA increased the plasma concentration of Met, but also the total sulfur AAs, including cysteine, which represent the key amino acids (**AAs**) in the production of glutathione and taurine within the trans-sulfuration pathway. The same study reported that, during the first 8 weeks of lactation, FOA supply increased milk production by 3.4 kg/d and the secretion of milk protein by 75 g/d, in accordance with previous results from the same research group [17]. 

During the transition period from late gestation to early lactation, it is usual for dairy cows to experience an imbalance between dietary energy, protein, and micronutrient intakes and nutrient demands for milk production, challenging the energy metabolism status and the immune system [18]. Several studies conducted by our group underscored the key role of one-carbon metabolism and Met supply in the modulation of the immunometabolic response, especially immediately after parturition [1,2,3]. Others also demonstrated that the supplementation of a mixture of cyanocobalamin, Met, and α-lipoic acid had positive effects on plasma biomarkers of liver and energy metabolism in postpartal dairy cows, underscoring their importance in reducing the risk of metabolic disorders [19]. Despite the fact that a number of studies have determined the effect of different Co sources (organic or inorganic), FOA, and vitamin B_12_ (alone or in combination) on lactation performance, energy status, and their content in plasma, liver, and milk, there are limited data available on the response of production performance together with the hepatic activity and mRNA abundance of key enzymes involved in the synthesis of Met, the trans-sulfuration pathway, and the immunometabolic status. 

Our hypothesis was that additional sources of one-carbon units from an increase in the dietary supply of Co, FOA, and Met (increasing vitamin B_12_ ruminal synthesis and methyl donors) would enhance the activity of the hepatic one-carbon metabolism, which, in turn, would result in a better immunometabolic status and performance. In addition, it was hypothesized that Co sources differing in the degree of ruminal availability to microbes may elicit different responses. Thus, the objective of the present study was to investigate the effects of supplementing two Co sources, an experimental source of FOA, and rumen-protected Met (**RPM**) on production performance, immunometabolic status, and hepatic one-carbon metabolism enzymes (mRNA abundance and enzyme activity) in peripartal Holstein cows.

## 2. Materials and Methods

### 2.1. Animal Management and Experimental Design

All procedures for this study were conducted in accordance with a protocol approved by the Institutional Animal Care and Use Committee at University of Illinois, Urbana-Champaign (protocol no. 17168, 2017). A total of 72 multiparous Holstein cows from the University of Illinois Dairy Research Farm were randomly allocated into 4 groups and blocked according to the expected calving date, and cows within each block were balanced for parity (2.96 ± 0.52), previous lactation milk yield (11,153 ± 1069 kg), and body condition score (BCS; 1-to-5 scale (1 = thin, 5 = fat) in increments of 0.25; 3.29 ± 0.12) before the close-up. From −45 to −30 d, cows were fed a typical Midwestern USA far-off diet, from −30 d to calving, cows received a close-up diet [1.37 Mcal/kg of DM and 14.5% crude protein (CP)], and, from calving until 30 days in milk (DIM), they received a lactation diet (1.65 Mcal/kg of DM and 17% CP) (Table 1). Samples of TMR were collected weekly, stored at –20 °C, and composited monthly. Composited samples were analyzed for DM, CP, ADF, NDF, starch, fat, Ca, P, Mg, K, Na, Cl, and S using wet chemistry methods at a commercial laboratory (Dairy One Cooperative Inc., Ithaca, NY, USA). Cows were fed a total mixed ration (TMR) once daily (07:00 h). Dry cows were housed in a free-stall barn with an individual Calan gate feeding system (American Calan, Northwood, NH, USA) and had access to sand-bedded free stalls. After calving, cows were housed in a tie-stall barn and were fed a common lactation TMR in their individual feed bunks once daily in the morning, and were milked 3 times daily (at 05:00, 13:00, 23:00 h). Dry matter intake (DMI) and milk yield were recorded daily. Diets were formulated to meet predicted requirements for dairy cows [20]. During the close-up and first 30 DIM, cows were supplemented with Co glucoheptonate (CoPro, 1 mol of Co bound to 2 mol of glucoheptonate; Zinpro Corp; n = 19), FOA plus Co (CoPro + FOA; n = 17), FOA plus Co pectin (1 mole of Co bound to 1 mole of pectin; CoPectin + FOA; Zinpro Corp, n = 18), or the combinations of FOA source, CoPectin, and RPM (CoPectin + FOA + RPM; Smartamine M was the source of RPM, Adisseo, Alpharetta, GA, USA; n = 18). In each FOA group, cows received 50 mg FOA/d. Cobalt treatments delivered 1 ppm Co/kg DM. The Co from CoPro would dissociate from Co glucoheptonate quickly and in a relatively short period of time, whereas the dissociation of Co from pectin would be slower and over a longer period of time. Thus, the latter would lead to a more sustained release of Co to ruminal microbes. The RPM was fed at 0.09% of DMI to achieve a ratio of 2.8:1 Lys:Met in the metabolizable protein (MP).

### 2.2. Feed and Milk Sampling

Feed offered and refused were measured daily for each cow. Feed samples for each ingredient were dried weekly to determine the DM and the rations were adjusted according to DM ratios of ingredients in the TMR. Samples of ingredients and TMR were taken weekly and frozen at −20 °C and composited monthly for further analysis. For contents of fat, protein, lactose, solids-not-fat (SNF), milk urea N (MUN), and somatic cell count (SCC), milk samples were collected from individual cows thrice weekly from morning, mid-day, and evening milking until 30 DIM, composited in proportion to milk yield at each milking, and preserved with preservative to prevent microbial growth (800 Broad Spectrum Microtabs II; D & F Control Systems Inc., San Ramon, CA, USA) prior to shipment to a commercial laboratory (Dairy Lab Services, Dubuque, IA, USA).

### 2.3. Blood Collection and Analysis

Blood was sampled from the coccygeal vein using vacutainer tubes containing lithium heparin (BD Vacutainer, BD and Co., Franklin Lakes, NJ, USA) and placed on ice in the morning before cows had access to feed at −30, −10, 7, 15, and 30 d relative to calving. Plasma was obtained by centrifugation at 2000× *g* for 15 min at 4 °C and aliquots were stored at −80 °C until further analysis. A clinical auto-analyzer (ILAB-650, Instrumentation Laboratory Werfen) was used to determine concentrations of glucose, β-hydroxybutyrate, free fatty acids, urea, cholesterol, albumin, haptoglobin, ceruloplasmin, paraoxonase, γ-glutamyl transferase (GGT), aspartate aminotransferase (AST/GOT), alkaline phosphatase, total bilirubin, Zn, reactive oxygen metabolites, myeloperoxidase, ferric reducing antioxidant power (FRAP), NOx, NO_2_^−^, and NO_3_^−^ [21,22,23]. Plasma retinol, tocopherol, and β-carotene were extracted with hexane and analyzed by reverse-phase HPLC using Spherisorb ODS-2, 3 lm, in a 150 × 4.6 mm column (Alltech, Deerfield, IL, USA); a UV detector set at 325 (for retinol), 290 (for tocopherol), or 460 nm (for β-carotene); and 80:20 methanol:tetrahydrofuran as the mobile phase.

### 2.4. Liver Biopsy and Tissue Analyses

Liver tissue from 12 cows among those used for plasma biomarker analyses that had the complete set of biopsies at −15, 7, 15, and 30 d relative to calving was harvested using published procedures [1,24]. Briefly, the skin was shaved and disinfected, and the skin and body wall were anesthetized with 7 mL of 2% lidocaine HCL (VetOne, Boise, ID, USA). A stab incision was made through the skin in the right 11th intercostal space, through which an 18-gauge by 10.2 cm bone marrow probe (Monoject-8881-247087; Medtronic, Minneapolis, MN, USA) was inserted into the liver and used to obtain approximately 4 g (wet weight) of liver. No more than three separate penetrations with the biopsy probe were performed. Samples were snap-frozen in liquid nitrogen and subsequently stored at −80 °C. 

All RNA extraction procedures were performed as described previously [25]. Briefly, RNA was extracted from liver samples using the miRNeasy kit (Qiagen, Hilden, Germany) following the manufacturer’s protocol. To remove genomic DNA from the RNA, samples were treated in column with DNaseI (Qiagen). NanoDrop ND-1000 (NanoDrop Technologies, Rockland, DE, USA) was used to measure RNA concentration, and RNA quality was measured using an Agilent 2100 Bioanalyzer (Agilent, Santa Clara, CA, USA). All samples had an RNA integrity number factor greater than 8. RNA was diluted to 100 ng with DNase/RNase-free water for cDNA synthesis by using RT-PCR, and the cDNA was diluted 1:4 with DNase/RNase-free water. The quantitative PCR (qPCR) performed was SYBR-Green-based (Quanta Biosciences, Beverly, MA, USA) using a 7-point standard curve obtained from a cDNA pool of all samples. Genes under investigation selected for transcript analysis were betaine–homocysteine methyltransferase (**BHMT**), betaine aldehyde dehydrogenase (**BADH**), cystathione beta-synthase (**CBS**), choline dehydrogenase (**CHDH**), dimethylglycine dehydrogenase (**DMGDH**), methionine adenosyltransferase 1A (**MAT1A**), methylenetetrahydrofolate reductase (**MTHFR**), methionine synthase (**MTR**), methionine synthase reductase (**MTRR**), phosphatidylethanolamine N-Methyltransferase (**PEMT**), sarcosine dehydrogenase (**SARDH**), S-adenosylhomocysteine hydrolase (**SAHH**), and methylmalonyl-CoA mutase (**MUT**). Primer information and primer sequencing results are included in Supplementary Table S1.

Activities of BHMT, CBS, and methionine adenosyltransferase (MAT) were determined according to protocols detailed by Coleman et al. [26] and Zhou et al. [27]. In addition, total protein concentration in these samples was measured via the Bradford assay (no. 500–0205, Bio-Rad Laboratories Inc., Hercules, CA, USA).

### 2.5. Statistical Analysis

Statistical analysis was performed with SAS 9.4. Data were subjected to ANOVA using repeated-measures ANOVA with PROC MIXED, including treatment (Trt), time (Time), and treatment × time (Trt × Time) as fixed effects, whereas cow was the random effect. For blood biomarkers, data at −30 days relative to parturition were used as covariate and four structures of covariance (compound symmetry, autoregressive order, Toeplitz, or spatial power) were tested in order to retain in the model the one with the lowest Akaike information criterion. For all data, the Kenward–Roger statement was used for computing the denominator degrees of freedom. Normality of the residuals was checked with normal probability and box plots, and homogeneity of variances was checked with plots of residuals versus predicted values. The geometric mean of the 3 internal control genes (*UXT*, *GAPDH*, and *RPS9*) was used to normalize the real-time quantitative PCR data and log2 transformed before statistical analysis to obtain a normal distribution. Data were considered significant at *p* ≤ 0.05 and tendencies at 0.05 < *p* ≤ 0.10, using the PDIFF statement in SAS. In addition, when a significant interaction was identified, the LSMEANS statement in SAS was used to compare means at each time point.

## 3. Results and Discussion

### 3.1. Dry Matter Intake and Milk Production

**Figure 1:**
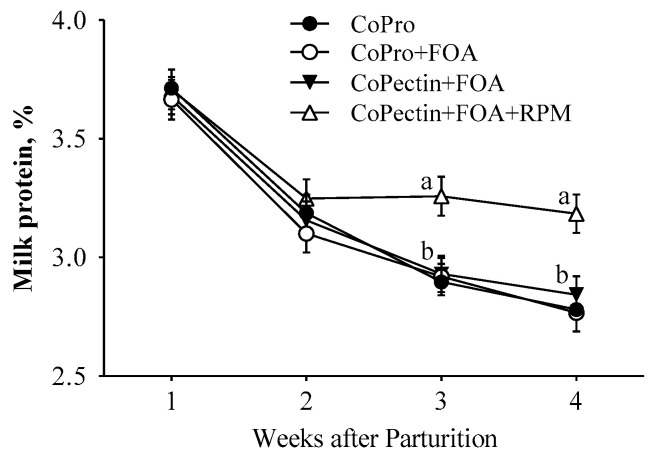
Milk protein percentage in multiparous Holstein cows fed 2 different Co sources [Co glucoheptonate (CoPro) or Co pectin (CoPectin)], folic acid (FOA), and rumen-protected methionine (RPM) during the close-up (from −30 d to parturition) and early lactation (from 1 to 30 days in milk) periods. Groups were CoPro, CoPro + FOA, CoPectin + FOA, and CoPectin + FOA + RPM. ^a–b^ Different letters indicate significant differences among treatments within a time point (*p* ≤ 0.05).

During the prepartal period, the DMI was not affected by the supplementation of different Co sources, FOA, or the combination of FOA, CoPectin, and RPM (*p* > 0.05; Table 2). However, as expected, the DMI of cows in all treatments decreased gradually (*p* < 0.01; Table 2) from −20 d to calving. Similar to prepartum, postpartal DMI was not affected by any of the treatments. A significant (*p* < 0.01; Table 2) increase in postpartal DMI was detected due to the increase in DMI in all groups after the first week of lactation. The main effects of treatment, time, and their interactions for milk composition and milk production variables are presented in Table 2. A significant interaction (Trt × Time; *p* = 0.02; Figure 1) was detected for milk protein percentage due to the greatest values occurring after the second week of lactation in the CoPectin + FOA + RPM group compared with other groups (*p* < 0.01). 

Previous studies with Co and folate supplementation have also reported no differences in milk yield and DMI [12,13,17,28]. Unlike previous findings, however, the present study considered the evaluation of different Co sources as the main objective rather than Co supplementation, and in combination with FOA and RPM. Thus, a direct comparison with previous similar studies is not straightforward. However, to assess effects with the FOA treatment, the CoPro group can be considered as the control relative to CoPro + FOA and differences between CoPro + FOA and CoPectin + FOA are strictly discussed with data generated in the present study without speculating relative to published studies. The only effect obtained at the milk level was that of RPM on protein percentage, with its increase at week 3 and 4 of lactation. A greater milk protein content is a well-established outcome when dairy cows are fed RPM during the transition period or during mid-lactation. In fact, these data are in line with those reported previously by our group [2,3,29]. Similarly, from a meta-analysis, Patton [30] speculated that Met-deficient dairy cows responded to RPM supply with a greater milk protein and fat content. 

This result underscores the fact that milk protein early postpartum is directly affected by adequate AA balance in the MP reaching the small intestine [20]. Especially in the early stage of lactation compared with other stages of lactation, when energy and protein intake cannot cope with the requirements from an increased milk production, the fact that RPM was able to increase milk protein content is noteworthy. Mechanistically, Met is identified frequently in dairy cows as a signaling factor that regulates protein synthesis in the mammary gland through the mechanistic target of rapamycin complex 1 (mTORC1) [31]. Indeed, recently, it was reported that a greater supply of Met in cultures of bovine mammary epithelial cells (reaching the ideal profile of AA in the Lys:Met ratio close to ~3:1) increased both the mRNA and protein abundance of α_S1_-casein and β-casein positively, and had a stimulatory effect on total and phosphorylated mTOR [32].

### 3.2. Blood Biomarkers and the Hepatic One-Carbon Metabolism Pathway

**Figure 2:**
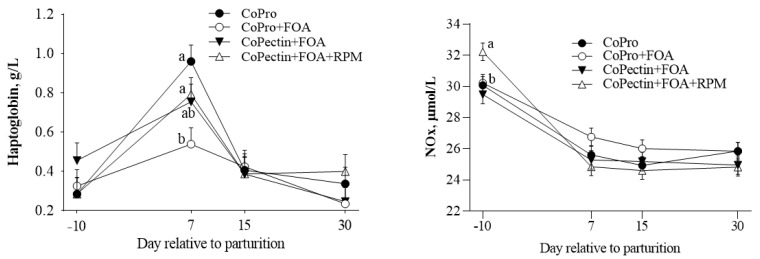
Plasma haptoglobin levels in multiparous Holstein cows fed 2 different Co sources [cobalt glucoheptonate (CoPro) or Co pectin (CoPectin)], folic acid (FOA), and rumen-protected methionine (RPM) during the close-up (from −30 d to parturition) and early lactation (from 1 to 30 days in milk) periods. Groups were CoPro, CoPro + FOA, CoPectin + FOA, and CoPectin + FOA + RPM. ^a–b^ Different letters indicate significant differences among treatments (*p* ≤ 0.05).

The responses in plasma concentrations of biomarkers related to energy metabolism, inflammation, liver function, and antioxidant status are presented in Table 3. No main effect was detected in the concentrations of these biomarkers. A tendency for the interaction Trt × Time was detected for haptoglobin (Trt × Time; *p* = 0.07), where, at day 7, CoPro + FOA cows had lower values compared with CoPro and CoPectin + FOA + RPM (*p* < 0.01; Figure 2), whereas no differences were detected among CoPro, CoPectin + FOA, and CoPectin + FOA + RPM. Lastly, a Trt × Time interaction (*p* = 0.01; Figure 2) was detected for nitric oxide metabolites due to a greater concentration at −10 d from parturition in cows that received CoPectin + FOA + RPM compared with other treatments.

The changes in blood haptoglobin from −10 to 7 d, especially between CoPro and CoPro + FOA, suggested a role of FOA supply in mitigating the inflammatory response after calving. However, the fact that the CoPectin groups did not seem to benefit from FOA is noteworthy: first, because it may suggest that they experienced a greater degree of inflammation (at least transiently) and, second, because they had greater hepatic MAT activity (and potentially flux through the Met cycle), especially postpartum (discussed below). As in humans, it could be speculated that FOA supplementation (in its role as a cofactor for one-carbon transfer reactions) could positively impact the production of antioxidants through the trans-sulfuration pathway, lowering the inflammatory response after parturition. Clearly, FOA can directly impact the synthesis of DNA nucleotides, concentrations of the amino acid Met, and the regulation of homocysteine levels. Emerging evidence suggests that these reactions have roles in the modulation of inflammatory responses via two different actions: (1) the remethylation of homocysteine to form Met, thus regulating the level of homocysteine, which, at elevated levels, may elicit an inflammatory response, and (2) the synthesis of glutathione and taurine, which are antioxidants, through the entry of homocysteine in the trans-sulfuration pathway as a consequence of a greater flux of Met through MAT [1,6,33,34]. Although the cows in the present study did not have clinical signs of disease, a reduction in taurine availability was recently linked with hyperketonemia postpartum in water buffalo [35]. 

As suggested by the clear decrease in concentrations of nitric oxide metabolites after calving regardless of treatment, the greater concentration in cows fed CoPectin + FOA + RPM at −10 d relative to calving did not seem pathological. In fact, this temporal pattern in NOx is similar to what we observed in a previous study, where CoPro along with organic Zn, Mn, and Cu (Availa^®^Zn, Availa^®^Mn, Availa^®^Cu; Zinpro Corp.) replaced a portion of the total inorganic trace mineral supplement from −30 to 30 d around parturition [36]. In another study dealing with feeding RPM during the same period around transition, we detected lower prepartal NOx concentrations due to RPM, with a temporal decrease after calving, where diet had no effect on concentrations [37]. The fact that greater concentrations of nitrate (NO_3_) and not NOx were associated with postpartal ketosis (i.e., ≥1.4 mmol β-hydroxybutyrate/L) [38] supports the idea that the observed differences in NOx prepartum are not pathological. 

**Figure 3:**
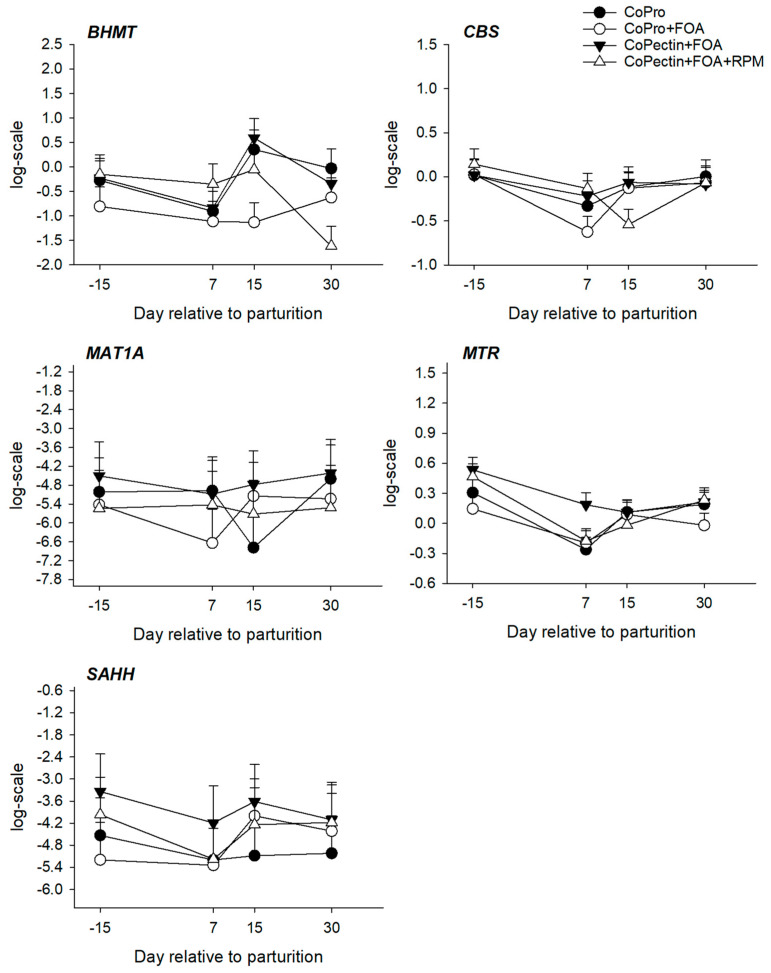
Hepatic mRNA abundance of genes encoding the key enzymes in the Met cycle, folic acid cycle, and trans-sulfuration pathway: methionine adenosyltransferase 1A (*MAT1A*), betaine–homocysteine S-methyltransferase (*BHMT*), cystathionine beta-synthase (*CBS*), and methionine synthase (*MTR*) in multiparous Holstein cows fed 2 different Co sources [Co glucoheptonate (CoPro) or Co pectin (CoPectin)], folic acid (FOA), and rumen-protected methionine (RPM) during the close-up (from −30 d to parturition) and early lactation (from 1 to 30 days in milk) periods. Groups were (n = 12 cows/group) CoPro, CoPro + FOA, CoPectin + FOA, and CoPectin + FOA + RPM. The *p* value for time effects was significant for all genes except *BHMT* (*p* = 0.08) and *MAT1A* (*p* = 0.20).

**Figure 4:**
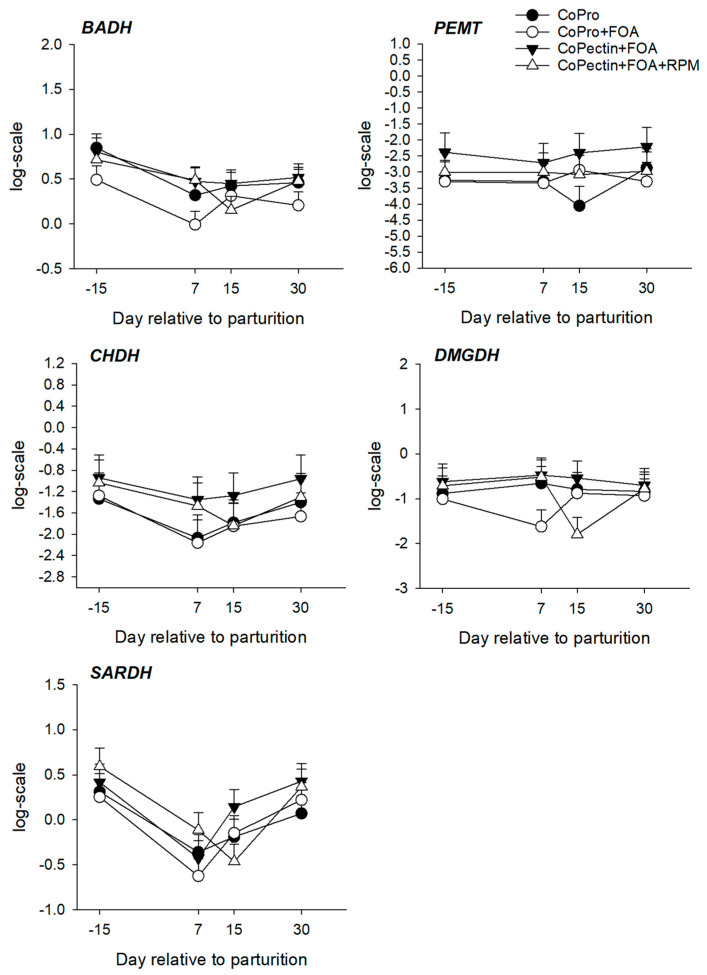
Hepatic mRNA abundance of genes encoding enzymes related to choline metabolism: betaine aldehyde dehydrogenase (*BADH*), phosphatidylethanolamine N-methyltransferase (*PEMT*), choline dehydrogenase (*CHDH*), and dimethylglycine dehydrogenase (*DMGDH*) in multiparous Holstein cows fed 2 different Co sources [Co glucoheptonate (CoPro) or Co pectin (CoPectin)], folic acid (FOA), and rumen-protected methionine (RPM) during the close-up (from −30 d to parturition) and early lactation (from 1 to 30 days in milk) periods. Groups were (n = 12 cows/group) CoPro, CoPro + FOA, CoPectin + FOA, and CoPectin + FOA + RPM. The *p* value for time effects was significant for all genes except *DMGDH* (*p* = 0.83) and *PEMT* (*p* = 0.60).

**Figure 5:**
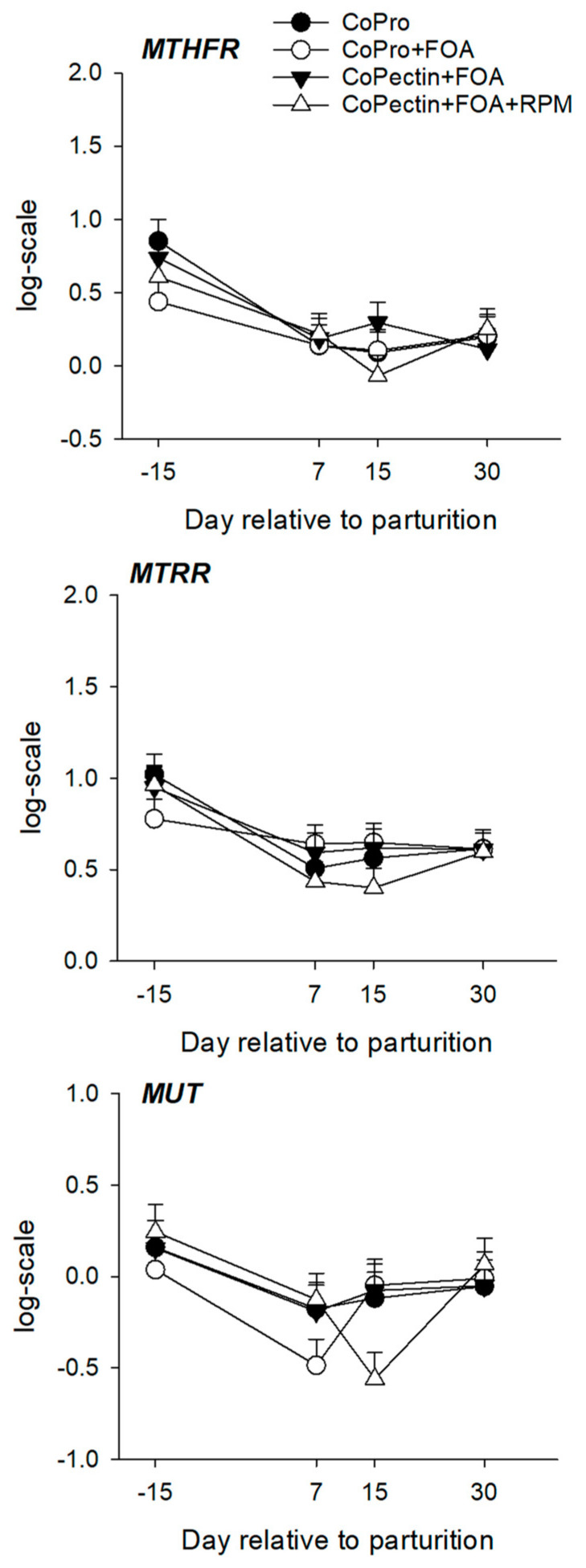
Hepatic mRNA abundance of genes encoding enzymes associated with riboflavin and cobalamin metabolism: methylenetetrahydrofolate reductase (*MTHFR*), 5-methyltetrahydrofolate-homocysteine methyltransferase reductase (*MTRR*), methylmalonyl-CoA mutase (*MMUT*), and sarcosine dehydrogenase (*SARDH*) in multiparous Holstein cows fed 2 different Co sources [Co glucoheptonate (CoPro) or Co pectin (CoPectin)], folic acid (FOA), and rumen-protected methionine (RPM) during the close-up (from −30 d to parturition) and early lactation (from 1 to 30 days in milk) periods. Groups were (n = 12 cows/group) CoPro, CoPro + FOA, CoPectin + FOA, and CoPectin + FOA + RPM.

**Figure 6:**
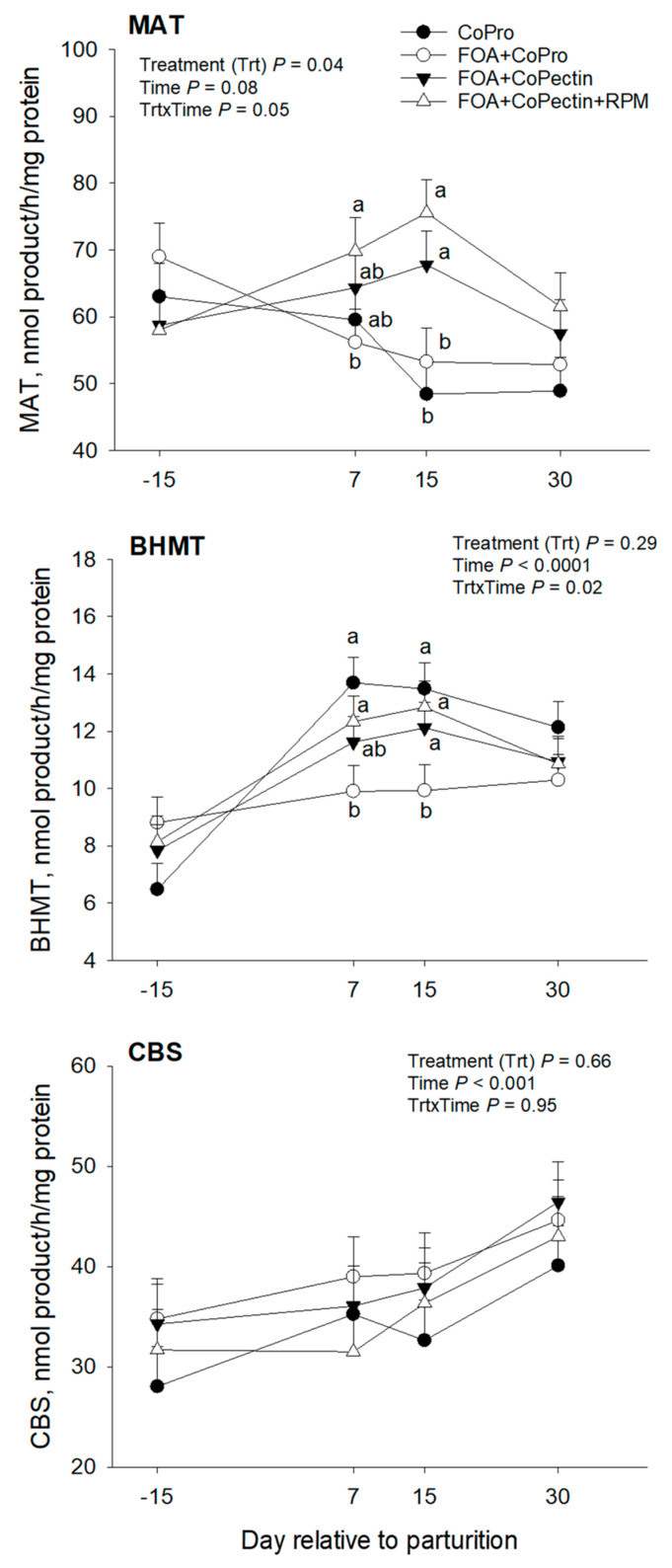
Hepatic activities of the one-carbon metabolism enzymes methionine adenosyltransferase 1 (MAT), betaine–homocysteine S-methyltransferase (BHMT), and cystathionine beta-synthase (CBS) in multiparous Holstein cows fed 2 different Co sources [Co glucoheptonate (CoPro) or Co pectin (CoPectin)], folic acid (FOA), and rumen-protected methionine (RPM) during the close-up (from −30 d to parturition) and early lactation (from 1 to 30 days in milk) periods. Groups were (n = 12 cows/group) CoPro, CoPro + FOA, CoPectin + FOA, and CoPectin + FOA + RPM. ^a–b^ Different letters indicate significant differences among treatments within a time point (*p* ≤ 0.05).

Hepatic mRNA abundance for genes within the one-carbon metabolism pathway are summarized in Table 4 and Figure 3, Figure 4 and Figure 5, whereas profiles for the activity of BHMT, MTR, and MAT are depicted in Figure 6. Overall, except for *BHMT*, there were no main effects of diet or interactions for the genes studied. Except for the mRNA abundance of *DMGDH*, *PEMT*, *MAT1A*, and *SAHH*, there was a significant (*p* < 0.02) effect of time on mRNA abundance for other genes. Feeding CoPectin + FOA led to a 1.6-fold greater overall (*p* = 0.03) *BHMT* abundance compared with CoPro + FOA, especially due to differences at 15 days. The activity of hepatic MAT, leading to the production of SAM, was greater (Trt, *p* = 0.04) for CoPectin + FOA or CoPectin + FOA + RPM than CoPro or CoPro + FOA. Specifically, at day 7 after parturition, cows in the CoPectin + FOA + RPM group had greater hepatic MAT activity compared with cows in the CoPro + FOA group (Trt × Time, *p* < 0.05; Figure 6). In contrast, no differences were detected among CoPro, CoPro + FOA, and CoPectin + FOA (Trt × Time, *p* > 0.10; Figure 6). At the same time point, there were also no differences observed between CoPro and CoPro + FOA, resulting in the lowest activity (Trt × Time, *p* > 0.10; Figure 6). At day 15 after parturition, instead, in view of the fact that, from day 7 to day 15, hepatic MAT activity increased in the CoPectin + FOA or CoPectin + FOA + RPM groups and decreased in the CoPro or CoPro + FOA, the former had greater MAT activity compared with the latter groups (Trt × Time, *p* < 0.05; Figure 6).

Overall, the activity of hepatic BHMT, a key enzyme for the transfer of methyl groups from betaine to homocysteine forming Met (remethylation), was greater in the postpartum compared with the late dry period (Time, *p* < 0.01; Figure 6). At day 7 after calving, cows receiving CoPro + FOA had the lowest values of BHMT activity, differing from the CoPro or CoPectin + FOA + RPM group (Trt × Time, *p* < 0.01), but not from the CoPectin + FOA group (Trt × Time, *p* > 0.10; Figure 6). At day 15, instead, the CoPro + FOA group had lower values of BHMT activity compared with CoPro, CoPectin + FOA, or CoPectin + FOA + RPM (Trt × Time, *p* < 0.01, Figure 6).

Folic acid, betaine, and Met are important nutrients that participate in one-carbon metabolism [6]. Besides the remethylation of homocysteine to Met using betaine or folate as methyl donors via BHMT and MTR, the enzyme MAT converts Met to SAM, the major cellular methyl donor that, in part, furnishes a transmethylation reaction catalyzed by phosphatidylethanolamine methyltransferase to generate S-adenosylhomocysteine (SAH) and phosphatidylcholine [6]. This is followed by the conversion of SAH to homocysteine in a reversible reaction catalyzed by SAH hydrolase. Homocysteine may also enter the trans-sulfuration pathway, the first reaction of which is used to synthesize cystathionine via the rate-limiting enzyme CBS. Cystathionine can then be used to make cysteine, which is utilized to synthesize the antioxidants glutathione and taurine [5,6]. The latter is also used to produce bile acids, which are an important aspect of cholesterol homeostasis in the liver.

Herein, hepatic MAT activity, especially at 7 days after calving, was greater in cows fed CoPectin + FOA or CoPectin + FOA + RPM, with the latter having numerically greater values than CoPro or CoPro + FOA cows. Even though hepatic or plasma levels of vitamin B_12_ were not measured, it is possible that this response was due to the Co complexed with pectin being more available for the ruminal synthesis of vitamin B_12,_ and, as a consequence, a greater flux of this vitamin toward folate-mediated reactions occurring. The same trend was also observed for cows that received RPM and, since in those cows the levels of MAT activity were numerically greater than CoPectin + FOA, it can be speculated that, in addition to the potentially greater production of methyl groups derived from 5-methyltetrahydrofolate, there would also be a greater flux of Met when its dietary supply increases in the form of RPM.

It is noteworthy to report that the study of Preynat et al. [39] demonstrated an increase in plasma Met concentrations with the combined supplementation of FOA and vitamin B_12_ in lactating cows not supplemented with RPM, reaching levels similar to those in cows fed RPM at 18 g/d/head. The responses observed for the activity and mRNA abundance of BHMT in the present study suggested that the preferential pathway for the donation of methyl groups affected by the Co, FOA, and RPM supply is the folate-MTR reaction. This idea is in line with the fact that the betaine pathway for the remethylation of homocysteine does not need the involvement of folate and vitamin B_12_.

## 4. Conclusions

Dietary supplements of different Co sources (glucoheptonate and pectin), folic acid, and RPM during the transition period did not affect DMI (pre and postpartum) or milk yield. However, data confirmed the role of feeding RPM, in order to achieve a Lys:Met ratio of ~2.9:1 in the metabolizable protein, for enhancing milk protein. Although the lack of vitamin B_12_ quantification at the liver and plasma levels is a limitation of the present study, the mRNA abundance and activity of BHMT indicated that supplying Co complexed with pectin or Co pectin plus RPM may enhance remethylation after parturition via the folate–methionine synthase pathway. As such, Met flux through the Met cycle might increase and enhance the availability of *S*-adenosylmethionine via MAT activity, thus increasing the overall methyl group supply within the liver.

## Figures and Tables

**Table 1:** Ingredient and nutrient composition of close-up (from −30 d to calving) and early lactation (from calving to 30 d) diets.

	Diets	
Item	Close-Up	Lactation
Ingredient (% of DM)		
Corn silage	37.47	41.12
Ground shelled corn	11.60	23.95
Wheat straw	24.00	2.30
Canola meal	11.67	3.25
Soybean meal	6.30	13.12
Alfalfa hay	-	8.70
Soyhulls	-	0.36
Soychlor^® 1^	3.37	-
Corn gluten feed	2.80	2.50
Mineral–vitamin mix	1.19	3.26
ProvAAL2 AADvantage^® 2^	0.47	0.73
Biotin ^3^	0.10	0.08
AjiPro-L^® 4^	0.06	0.06
Availa^®^ Dairy ^5^	0.05	0.06
Rumensin^® 6^	0.19	0.02
Calcium sulfate	0.53	0.12
Magnesium oxide	0.10	0.12
Salt	0.10	0.25
Chemical composition		
DM, %	42.88	47.26
CP, % of DM	14.60	17.00
NDF, % of DM	39.15	21.50
ADF, % of DM	31.08	16.76
NFC, % of DM	27.68	46.83
Starch, % of DM	17.17	29.46
Crude fat, % of DM	2.53	2.74
NE_L_, Mcal/kg of DM	1.37	1.65
NE_L_ allowable milk, kg/d	-	37.82
MP allowable milk, kg/d	-	40.55
RDP, % of DM	8.92	10.54
RUP, % of DM	5.68	6.46
RDP required, g/d	1061	2435
RDP supplied, g/d	1116	2581
RDP balance, g/d	49	145
RUP required, g/d	168	1043
RUP supplied, g/d	676	1583
RUP balance, g/d	503	540
MP required, g/d	776	2341
MP supplied, g/d	1188	2803
MP balance, g/d	413	463
Ca	0.66	1.00
P	0.33	0.35
Na	0.12	0.45
Cl	0.78	0.68
Mg	0.45	0.38
K	1.36	1.45
S	0.33	0.20

^1^ Landus (Des Moines, IA, USA); ^2^ Perdue AgriBusiness (Salisbury, MD, USA); ^3^ ADM Animal Nutrition (Quincy, IL, USA); ^4^ Ajinomoto Health & Nutrition North America, Inc. (Itasca, IL, USA); ^5^ Zinpro Corp. (Eden Prairie, MN, USA); ^6^ Elanco Animal Health (Greenfield, IN, USA).

**Table 2:** Dry matter intake (DMI) and milk performance in multiparous Holstein cows fed 2 different Co sources [Co glucoheptonate (CoPro) or Co pectin (CoPectin)], folic acid (FOA), and rumen-protected methionine (RPM) during the close-up (from −30 d to parturition) and early lactation (from 1 to 30 days in milk) periods. Groups were CoPro, CoPro + FOA, CoPectin + FOA, and CoPectin + FOA + RPM.

Item	Treatment				SEM ^1^	*p*-Value		
	CoPro	CoPro + FOA	CoPectin + FOA	CoPectin + FOA + RPM		Trt	Time	Trt × Time
Total, no.	19	17	18	18				
Close-up period								
DMI, kg/d	12.9	13.0	13.2	13.3	0.6	0.98	<0.01	0.24
Fresh period								
DMI, kg/d	15.9	15.8	15.9	15.9	0.8	0.99	<0.01	0.99
Milk, kg/d	40.7	39.0	39.7	40.8	1.9	0.87	<0.01	0.25
Body condition score	3.01	3.02	2.99	2.99	0.10	0.99	<0.01	0.46
Milk composition, %								
Fat	3.65	3.65	3.62	3.94	0.16	0.41	<0.01	0.40
Protein	3.14 ^b^	3.11 ^b^	3.15 ^b^	3.35 ^a^	0.07	0.08	<0.01	0.02
Lactose	4.82	4.86	4.83	4.85	0.04	0.87	<0.01	0.81
Total solids	12.5	13.4	11.9	11.9	0.76	0.48	0.18	0.31
SCC ^2^ × 1000	57.8	36.1	51.1	35.9	15.2	0.48	0.31	0.56
MUN ^3^, mg/dL	12.0	12.1	12.5	11.8	0.50	0.83	0.05	0.68

^1^ Greatest SEM; ^2^ Somatic cell count data were log-transformed; ^3^ Milk urea nitrogen. ^a–b^ Different superscripts indicate significant differences among treatments (*p* ≤ 0.05).

**Table 3:** Plasma biomarkers related to energy balance, inflammation, and oxidative stress in multiparous Holstein cows fed 2 different Co sources [Co glucoheptonate (CoPro) or Co pectin (CoPectin)], folic acid (FOA), and rumen-protected methionine (RPM) during the close-up (from −30 d to parturition) and early lactation (from 1 to 30 days in milk) periods. Groups were CoPro, CoPro + FOA, CoPectin + FOA, and CoPectin + FOA + RPM.

Item	Treatment				SEM	*p*-Value		
	CoPro	CoPro + FOA	CoPectin + FOA	CoPectin + FOA + RPM		Trt	Time	Trt × Time
Total, no.	16	16	16	16				
**Energy metabolism**								
Glucose, mmol/L	3.95	3.92	3.97	3.97	0.08	0.96	<0.01	0.55
Fatty acids, mmol/L	0.69	0.62	0.58	0.70	0.07	0.57	<0.01	0.64
Urea, mmol/L	5.30	5.60	5.38	5.31	0.27	0.81	<0.01	0.37
β-hydroxybutyrate, mmol/L	0.82	0.77	0.65	0.66	0.09	0.44	<0.01	0.59
**Inflammation**								
Ceruloplasmin, µmol/L	2.95	2.95	3.04	2.91	0.12	0.88	<0.01	0.96
Albumins, g/L	35.8	35.9	35.8	35.6	0.51	0.97	0.02	0.85
Haptoglobin, g/L	0.50	0.38	0.46	0.47	0.05	0.42	<0.01	0.07
Myeloperoxidase, U/L	497	498	475	474	21.1	0.73	<0.01	0.62
Zn, µmol/L	12.6	12.5	12.7	12.7	0.67	0.99	<0.01	0.10
**Liver function**								
Cholesterol, mmol/L	2.77	2.95	3.17	3.08	0.18	0.40	<0.01	0.99
AST/GOT, U/L	103	111	110	104	6.81	0.76	<0.01	0.60
GGT, U/L	22.9	23.0	27.6	22.6	2.12	0.28	<0.01	0.89
Total bilirubin, µmol/L	5.67	4.10	4.38	4.80	0.58	0.21	<0.01	0.74
Alkaline phosphatase, U/L	58.5	51.7	57.3	56.1	6.65	0.88	<0.01	0.97
Paraoxonase, U/mL	82.0	85.5	84.9	73.8	4.43	0.18	<0.01	0.67
Retinol, µg/mL	27.2	32.1	30.1	30.5	2.16	0.39	<0.01	0.11
**Antioxidant status**								
ROMt, mg H_2_O_2_/dL	14.9	14.7	15.5	14.6	0.45	0.54	<0.01	0.99
NOx, µmol/L	26.6	27.2	26.2	26.6	0.39	0.34	<0.01	0.01
NO_2_, µmol/L	4.42	4.70	4.81	4.89	0.29	0.65	<0.01	0.10
NO_3_, µmol/L	22.2	22.5	21.4	21.7	0.38	0.17	<0.01	0.65
FRAP, µmol/L	125	123	129	128	4.22	0.72	<0.01	0.34
Tocopherol, µg/mL	3.29	3.64	3.62	3.87	0.24	0.41	<0.01	0.27
β-carotene, mg/dL	0.19	0.21	0.17	0.21	0.02	0.39	<0.01	0.99

**Table 4:** Hepatic mRNA abundance in multiparous Holstein cows fed 2 different Co sources [cobalt glucoheptonate (CoPro) or Co pectin (CoPectin)], folic acid (FOA), and rumen-protected methionine (RPM) during the close-up (from −30 d to parturition) and early lactation (from 1 to 30 days in milk) periods. Groups were CoPro, CoPro + FOA, CoPectin + FOA, and CoPectin + FOA + RPM. ^a–b^ Different superscripts indicate significant differences among treatments (*p* ≤ 0.05).

Gene ^1^	Treatment				SEM	*p*-Value		
	CoPro	CoPro + FOA	CoPectin + FOA	CoPectin + FOA + RPM		Trt	Day	Trt × Day
**Met and folic acid cycle and trans-sulfuration**								
*BHMT*	0.86 ^a^	0.53 ^b^	0.87 ^a^	0.69 ^ab^	0.11	0.03	0.08	0.12
*CBS*	0.93	0.87	0.94	0.90	0.07	0.88	0.01	0.39
*MAT1A*	0.02	0.02	0.04	0.02	0.02	0.90	0.20	0.37
*MTR*	1.06	1.00	1.20	1.09	0.07	0.19	<0.01	0.22
*SAHH*	0.03	0.04	0.07	0.05	0.04	0.82	0.09	0.81
**Choline metabolism**								
*BADH*	1.43	1.19	1.48	1.37	0.11	0.20	<0.01	0.53
*PEMT*	0.10	0.11	0.19	0.12	0.07	0.62	0.60	0.61
*CHDH*	0.32	0.30	0.46	0.38	0.11	0.63	<0.01	0.97
*DMGDH*	0.58	0.46	0.67	0.52	0.11	0.42	0.83	0.37
*SARDH*	0.97	0.95	1.10	1.07	0.10	0.56	<0.01	0.33
**Riboflavin and cobalamin metabolism**								
*MTHFR*	1.25	1.17	1.26	1.19	0.07	0.75	<0.01	0.52
*MTRR*	1.60	1.59	1.62	1.51	0.06	0.64	<0.01	0.66
*MMUT*	0.97	0.92	0.97	0.94	0.05	0.85	<0.01	0.16

^1^ *BHMT* = betaine–homocysteine methyltransferase; *BADH* = betaine aldehyde dehydrogenase; *CBS* = cystathionine beta-synthase; *CHDH* = choline dehydrogenase; *DMGDH* = dimethylglycine dehydrogenase; *MAT1A* = methionine adenosyltransferase 1A; *MTHFR* = methylenetetrahydrofolate reductase; *MTR* = 5-methyltetrahydrofolate-homocysteine methyltransferase; *MTRR* = methionine synthase reductase; *PEMT* = phosphatidylethanolamine methyltransferase; *SARDH* = sarcosine dehydrogenase; *SAHH* = S-adenosylhomocysteine hydrolase; *MMUT* = methylmalonyl-CoA mutase.

## Data Availability

Data available upon request from the authors.

## Supplementary Materials

The following supporting information can be downloaded at: https://www.mdpi.com/article/10.3390/ani13132107/s1.
