# Supplementary material for: Influence of Cobalt Source, Folic Acid, and Rumen-Protected Methionine on Performance, Metabolism, and Liver Tissue One-Carbon Metabolism Biomarkers in Peripartal Holstein Cows
Source: Animals (Basel). 2023 Jun 25;13(13):2107. doi: 10.3390/ani13132107 (PMC10339869; doi:10.3390/ani13132107)
Supplement: Supplementary file 1 [file animals-13-02107-s001.zip › animals-2412552-supplementary.pdf]

**Supplemental Table S1**. qPCR performance and relative mRNA abundance of genes measured in liver tissue of Holstein cows fed 2 different Co sources [Co glucoheptonate (CoPro) or Co pectin (CoPectin)], folic acid (FOA), and rumen-protected methionine (RPM) during the close-up (from −30 d to parturition) and early lactation (from 1 to 30 days in milk) periods.

| Gene[1] | Median Ct[2] | Median ΔCt[3] | Slope[4] | ($R^2$ )[5] | Efficiency[6] | Relative mRNA abundance[7] |
|---|---|---|---|---|---|---|
| *BHMT* | 19.74116 | -1.408 | -3.423 | 0.996 | 1.959 | 2.578 |
| *BADH* | 22.08621 | 0.953 | -3.305 | 0.988 | 2.007 | 0.515 |
| *CBS* | 22.42008 | 1.208 | -3.305 | 0.991 | 2.007 | 0.431 |
| *CHDH* | 26.82888 | 5.826 | -3.909 | 0.966 | 1.802 | 0.032 |
| *DMGDH* | 21.38861 | 0.191 | -3.288 | 0.994 | 2.014 | 0.875 |
| *MAT1A* | 24.23237 | 3.038 | -3.371 | 0.995 | 1.980 | 0.126 |
| *MTHFR* | 24.34020 | 3.144 | -2.932 | 0.994 | 2.193 | 0.085 |
| *MTR* | 24.53422 | 3.318 | -3.086 | 0.979 | 2.109 | 0.084 |
| *MTRR* | 25.18261 | 4.005 | -2.888 | 0.990 | 2.219 | 0.041 |
| *PEMT* | 24.35371 | 3.125 | -3.964 | 0.990 | 1.788 | 0.163 |
| *SARDH* | 22.62557 | 1.452 | -3.263 | 0.992 | 2.025 | 0.359 |
| *SAHH* | 23.21901 | 2.232 | -3.689 | 0.991 | 1.867 | 0.248 |
| *MMUT* | 20.31831 | -0.876 | -3.352 | 0.995 | 1.988 | 1.826 |

[1]*BHMT* = betaine homocysteine methyltransferase; *BADH*= Betaine-aldehyde dehydrogenase; *CBS*= cystathionine beta-synthase; *CHDH*= Choline dehydrogenase; *DMGDH*= dimethylglycine dehydrogenase; *MAT1A*= methionine adenosyltransferase 1A; *MTHFR*= methylenetetrahydrofolate reductase; *MTR*= 5-methyltetrahydrofolate-homocysteine methyltransferase; *MTRR*= methionine synthase reductase; *PEMT*= phosphatidylethanolamine methyltransferase; *SARDH*= Sarcosine dehydrogenase; *SAHH*= S-adenosylhomocysteine hydrolase; *MMUT*= methylmalonyl-CoA mutase.

**Supplemental Table S2**. Accession number, gene symbol, and forward and reverse primer sequences of genes analyzed in liver tissue of Holstein cows fed 2 different Co sources [Co glucoheptonate (CoPro) or Co pectin (CoPectin)], folic acid (FOA), and rumen-protected methionine (RPM) during the close-up (from −30 d to parturition) and early lactation (from 1 to 30 days in milk) periods.

| Gene[1] | Accession number | Forward primer | Reverse Primer |
|---|---|---|---|
| *BHMT* | NM_001011679 | GCT CTC CTC GTC CAT CCT CAT | CCG TTC TAG GAT GCC CTT CTT |
| *BADH* | NM_001045969 | CAC CTA CTG TCC TGC TAA | ATC ACC AAT CTG TCT CAC TAC |
| *CBS* | NM_001102000 | GCC ACC ACC TCT GTC AAA TTC | GGA CAG AAA GCA GAG TGG TAA CTG |
| *CHDH* | NM_001205564 | AAA CTG AGA AGT GCC AAC | ACG GAA GTC TTT AAT GTC A |
| *DMGDH* | NM_001205545 | GCCAAGTTTTCACCGCACAA | AGGTCAATCACCCCCACTCT |
| *MAT1A* | NM_001046497 | CAA GGG CTT TGA CTT TAA | CCG ACA TCC TCT TCA TTT |
| *MTHFR* | NM_001011685 | TTC AAC TAT GCT ACG GAC TT | CCT TCA GGT GCT TCA GAT |
| *MTR* | NM_001030298 | ATA CCG CCA ATG CCA AGG | ATG AGA CAC GCT GAT GAC AA |
| *MTRR* | NM_001030299 | GCTTTTTCCTCAGCAACCCC | TCCTCATCACCTCCAGGCAT |
| *PEMT* | NM_182989 | AAT TAC CAA GAG CAG AGG | AAT TAC CAA GAG CAG AGG |
| *SARDH* | NM_001193041 | GAG GAG GTG TCA GAT GAG | GCA GAC TGT GGA CTT AAT C |
| *SAHH* | NM_001034315 | CAA TGT CAA TGA CTC TGT | CTT GAT GCC ATC TAT GAG |
| *MMUT* | XM_019986008. | CTTGTTTGGCGTGTTCCATGT | ATGACCCTTTCAGCTGCCTC |

[1]*BHMT* = betaine homocysteine methyltransferase; *BADH*= Betaine-aldehyde dehydrogenase; *CBS*= cystathionine beta-synthase; *CHDH*= Choline dehydrogenase*;* *DMGDH*= dimethylglycine dehydrogenase*;* *MAT1A*= methionine adenosyltransferase 1A; *MTHFR*= methylenetetrahydrofolate reductase; *MTR*= 5-methyltetrahydrofolate-homocysteine methyltransferase; *MTRR*= methionine synthase reductase; *PEMT*= phosphatidylethanolamine methyltransferase; *SARDH*= Sarcosine dehydrogenase*;* *SAHH*= S-adenosylhomocysteine hydrolase; *MUT*= methylmalonyl-CoA mutase.
